# Supplementary material for: The familiarity effect of Chinese stroke stimulus and imagery on contextual integration: Evidence from ERP correlates
Source: PLoS One. 2025 Jul 1;20(7):e0327138. doi: 10.1371/journal.pone.0327138 (PMC12212528; doi:10.1371/journal.pone.0327138)
Supplement: Attachment — Submitted filename: PONE-D-25-00635_Response to Reviewers_v02.docx [file pone.0327138.s005.docx]

Title: The Familiarity Effect of Chinese Stroke Stimulus and Imagery on Contextual Integration: Evidence from ERP Correlates [PONE-D-25-00635]

Sam C. C. Chan, Tom C. W. Tsoi

Response to Reviewers

|  | Comments | Responses (Line numbers as shown in the Revised Manuscript with Track Changes | remark |
| --- | --- | --- | --- |
| Editor’s Comment | | | |
|  | Please provide a power analysis to address this. | Thanks for the suggestion. Power analysis has been conducted and  Sample estimation is reported in the Materials and Methods section (Section 2.2). |  |
|  | In addition, please also state the number of items you have used in stimuli (currently mentioned in paradigm: 4 blocks of 16 trials per block. But it is unclear whether there were repetitions). Both pieces of information are important to evaluate the generalizability of your findings. | Number of Chinese characters used is included in the paradigm section (Section 2.4, Line 315 - 317). |  |
| Reviewer #1 | | | |
| 1.1 | The manuscript presents a technically sound study with a clear hypothesis and experimental design. The use of ERP components (N160, P200, N400) to investigate attentional control and contextual integration in processing familiar vs. unfamiliar Chinese strokes is well-grounded in prior literature. The tasks (imagery vs. detection) and stimuli (semi-cursive vs. regular styles) are appropriately designed to address the research questions. The study addresses an underexplored topic (sub-character-level contextual integration) using a novel paradigm with Chinese strokes. The integration of ERP components to dissociate attentional and semantic processes is methodologically robust. | Thanks for your valuable comments. |  |
| 1.2 | The study offers valuable insights into contextual integration mechanisms but requires methodological refinements, statistical transparency, and language improvements to meet publication standards.  Theoretically, the authors may discuss how findings extend beyond orthographic processing to general mechanisms of contextual integration (e.g., cross-modal comparisons). | Thanks for the comments. The Section 2.2 Sample Size Estimation, and the statistical details are added in the section 3 of Result.  Generalization / implications are explained in the discussion section (Line 632 - 646)  The manuscript has been copyedited. |  |
| 1.3a | The sample size (N=19) is relatively small for ERP studies, which may limit generalizability. While the results align with the hypotheses (e.g., larger N400 amplitudes for unfamiliar stimuli), the lack of behavioral data (e.g., accuracy, reaction times) to corroborate ERP findings weakens the conclusions. | Thanks for the comments. Sample estimation is included in the Materials and Methods section (Section 2.2).  Besides, the p-values reported in the results (Section 3.2 – 3.4) were relatively small, implying sufficient sample power. |  |
| 1.3b | The statistical analysis is generally appropriate, employing three-way repeated-measures ANOVA with Greenhouse-Geisser correction for sphericity violations. Post-hoc tests with Bonferroni adjustments are justified. | Thanks for the comments |  |
| 1.3c | However, critical details are missing:  1）The exact p-values for ANOVA results (e.g., p<0.005 for N160) are inconsistently reported. | Exact p-values are reported in the Section 3.2 – 3.4 of the Results section. |  |
| 1.3d | 2）Effect sizes (e.g., η²) are not provided, making it difficult to assess the magnitude of observed effects. | The values of effect size (η²) are reported in Section 3.2 – 3.4 of the Results section. |  |
| 1.3e | 3）The rationale for selecting midline electrodes (Fz, Cz, Pz) over other regions is not explicitly stated, raising concerns about potential cherry-picking. | The reasons to choose these three midline sites (Fz, Cz and Pz) are provided in Section 2.7 (Line 388 – 393). |  |
| 1.3f | 4）The manuscript does not address potential outliers or data normality assumptions.  These omissions reduce the rigor and transparency of the analysis. The author may want to justify the sample size (N=19) with a power analysis. Include demographic details (e.g., educational background, handedness) to contextualize findings. | Power analysis and Sample estimation is included in the Section 2.2 Sample Size Estimation.  Outlier analysis was added in Section 2.6 (Line 350 – 352). No outlier participants were identified  Offline preprocessing procedure was applied to reject outlying EEG data as described in Section 2.6 (Line 375 – 376).  Demographic details of the participants are included in Section 2.1 Participants as suggested (Line 257 – 260). |  |
| 1.3g | And also report accuracy and reaction times to validate ERP results and rule out task performance confounds. | The table of accuracy and reaction of the behavioral data during the experience (Table 1) are added as suggested (Line 428-431). The results are summarized in the main text (Section 3.1) |  |
|  | The writing is generally clear but contains grammatical errors and awkward phrasing that hinder readability. Thorough language editing is required to meet PLOS ONE’s standards.  1）Typos: “heighted” → “heightened” (Abstract, Line 4); “abd” → “and” (Page 12, Line 69).  2）Inconsistent terminology: “semi-cursive” vs. “Shinsu” (Page 19, Line 202); “radial-omitted” → “radical-omitted” (Page 14, Line 131).  3）Ambiguous sentences: E.g., “The education level of all selected characters was up to primary six” (Page 19, Line 208) – clarify if this refers to character complexity or participant education.  4）Formatting issues: Missing spaces (e.g., “N400 300-500ms)” → “N400 (300-500 ms)”), inconsistent citation styles (e.g., “Kutas & Federmeier, 2011” vs. “Kutas et al., 2011”). | The following revisions are made as suggested:  1）Typos: “heighted” has been revised to “heightened” (Abstract, Line 24) and “abd” has been revised to “and” (Line 100).  2） “Shinsu” is specified as the name of font used in the paradigm (Line 275)  “radial-omitted” has been amended to “radical-omitted” (Line 183).  The “education level” is replaced by the “character complexity” (Line 280)  “N400 300-500ms)” is changed to “N400 (300-500 ms)” (Line 34)  The citations are all standardized to be “Kutas & Federmeier” (Line 62, 158, 174, & 539) |  |
| Reviewer #2 | | | |
| 2.1 | This manuscript investigates how familiarity with Chinese stroke stimuli influences attentional processes and contextual integration, using ERP components (N160, P200, and N400) as neural markers. The focus on sub-character (stroke-level) processing is innovative and has the potential to contribute to our understanding of orthographic processing in Chinese. However, the study’s significance is undermined by a lack of clarity in its core concept—"incongruency" at the stroke level—as well as insufficient theoretical grounding, undefined terms, and methodological ambiguities. Addressing these issues through minor revisions will enhance the manuscript’s rigor and impact. | Thanks for the comments. Specific changes are made according to the recommendations in the next section |  |
| 2.2 | Major Concerns  1. Unclear Definition of "Incongruency" at the Stroke Level  The central concept of "incongruency" at the sub-character (stroke) level is not clearly defined, leaving readers uncertain about what it represents and why it matters. Specifically: | Thanks for the comments. Specific changes are made according to the recommendations in the next section |  |
| 2.3 | • The authors do not specify whether the incongruency pertains to stroke sequences during production (e.g., writing) or recognition (e.g., reading/perceiving). For example, if unexpected stroke sequences are presented in playback, this could reflect a production issue (deviating from how strokes are typically written) rather than a recognition issue (deviating from how strokes are expected in perception). Alternatively, it could involve both, but this ambiguity need to be addressed. The N400, typically linked to unexpected patterns or meanings, is used here to index stroke-level incongruency, but its relevance depends on whether the authors are targeting recognition, production, or a combination. |  |  |
| 2.4 | • The study appears to assume that stroke-level information (e.g., sequence or order) is stored and actively used in character recognition, making "incongruency" at this level detectable and meaningful. However, this assumption is neither explicitly stated nor supported with evidence. Without this foundation, the premise of studying stroke-level incongruency lacks justification. |  |  |
| 2.5 | • The authors frequently mention "integration," but it’s unclear how stroke-level incongruency relates to this process. If integration is about recognition (e.g., combining stroke information into a character), the design should test recognition-specific effects. If it involves production, this should be clarified. The current ambiguity makes it difficult to assess how stroke-level processing fits into the broader orthographic processing framework. |  |  |
| 2.6 | Recommendations:  • Explicitly define "incongruency" and specify whether it relates to production, recognition, or both. | Thanks for the recommendations.  The term “incongruency” is defined more clearly in terms of recognition  (Line 86 - 92) |  |
| 2.7 | • State the assumption that stroke-level information is stored and used in recognition (or production), and provide literature support (e.g., studies on stroke sequence encoding, sub-character processing, or orthographic integration in Chinese) | The assumption that the familiarity of stroke-level is stored and used in recognition is described as suggested (Line 97 - 109) |  |
| 2.8 | • Clarify how stroke-level incongruency contributes to orthographic integration, aligning it with the study’s research question. | How stroke-level incongruency would lead an increased effort for the contextual integration is explained more clearly (Line 102-111) |  |
| 2.9 | 2. Undefined Key Terms  Terms like "attention," "selective attention," "orientation of attention," and "integration" are used frequently but not defined. For instance, does "integration" refer to combining strokes into characters, contextual understanding, or something else? Brief definitions upon first use would improve clarity. | Definitions of “selective attention” and “attention orientation” are provided in section 1.1“ (Line 126-132)  Contextual integration” are explained in Line 111 - 122. |  |
| 2.10 | 3. Methodological concern  • The choice of midline sites (Fz, Cz, Pz) for ERP analysis is not explained. Why were these sites chosen for N160, P200, and N400 effects? | The reasons to choose the midline sites at Fz, Cz and Pz are provided in Section 2.7 (Line 388-393). |  |
| 2.11 | • Applying the N400—typically a semantic processing marker—to non-semantic stroke stimuli is less common. The authors need to provide precedent (e.g., N400 effects in orthographic or non-linguistic contexts) to support this approach. | The N400 has been found in the context of visual scenes, such as face and traffic light identification. (Section 1, Line 83-85) |  |
| 2.12 | Minor Concerns  • The assumption that semi-cursive strokes are unfamiliar to participants is untested. Evidence (e.g., a familiarity survey or participant background data) should confirm this, if this is difficult to achieve, author can consider providing support from literature | Inclusion criteria included asking participants whether they have prior training in Chinese calligraphy, especially in the semi-cursive style.  “(2) being native Chinese and being able to read Chinese; (3) not having prior formal training in Chinese calligraphy in semi-cursive style”. (Section 2.1, Line 250-256) |  |
| 2.13 | • The term "optimal balance" (related to attention) is vague. What does it mean, and how does it connect to the study’s goals? | “optimal balance” replaced by “effective”(line 148) |  |
| 2.14 | General Recommendation  This study’s focus on sub-character (stroke-level) processing is innovative and has the potential to advance understanding of orthographic processing in Chinese. However, the manuscript would benefit from clearer definition of "incongruency" (production vs. recognition), further clarification of assumptions regarding stroke-level storage, and additional theoretical and methodological explanations. I recommend minor revision to address these points: | Thanks for the comments. |  |
| 2.15 | • Define "incongruency" and clarify whether it pertains to production, recognition, or both, linking it to orthographic integration. | The term “incongruency” is defined more clearly in terms of recognition  (Line 86 - 92) |  |
| 2.16 | • Justify the assumption that stroke-level information is stored and used, with literature support. | The assumption that stroke-level is stored and used in recognition is described as suggested (Line 97-109) |  |
| 2.17 | • Improve theoretical rationale for studying stroke-level incongruency. | The theoretical rationale for studying stroke-level congruency is explained (Line 210-227) |  |
| 2.18 | • Define key terms (e.g., "attention," "integration"). | Definitions of “selective attention” and “attention orientation” are provided in section 1.1 (Line 126-132) |  |
| 2.19 | • Justify electrode sites and N400 use, and detail the experimental paradigm. | The reasons to choose these three midline sites (Fz, Cz and Pz) are provided in Section 2.7 (Line 388-393). |  |
| 2.20 | • Verify stimulus familiarity assumptions (or with support from literature). | The assumption on the stroke-level familiarity is described as suggested with literature support (Line 102-111) |  |
